# Navigated, percutaneous, three-step technique for lumbar and sacral screw placement: a novel, minimally invasive, and maximally safe strategy

**DOI:** 10.1186/s10195-023-00696-5

**Published:** 2023-06-29

**Authors:** Giuseppe La Rocca, Edoardo Mazzucchi, Fabrizio Pignotti, Luigi Aurelio Nasto, Gianluca Galieri, Pierluigi Rinaldi, Vincenzo De Santis, Enrico Pola, Giovanni Sabatino

**Affiliations:** 1 Department of Neurosurgery, Fondazione Policlinico Universitario “A. Gemelli” IRCCS, Catholic University of Rome School of Medicine, Rome, Italy; 2 Department of Neurosurgery, Mater Olbia Hospital, Olbia, Italy; 3 Department of Orthopaedics and Spine Surgery, Azienda Ospedaliera Universitaria “Luigi Vanvitelli”, Università Della Campania Luigi Vanvitelli, Via De Crecchio 4, 80138 Naples, Italy; 4 Department of Radiology, Mater Olbia Hospital, Olbia, Italy; 5 Department of Orthopaedics, Mater Olbia Hospital, Olbia, Italy

**Keywords:** Lumbo–sacral instrumentation, Percutaneous navigated screw placement, Minimally invasive spine surgery, Navigated drill guide

## Abstract

**Background:**

Minimally invasive spine surgery is a field of active and intense research. Image-guided percutaneous pedicle screw (PPS) placement is a valid alternative to the standard free-hand technique, thanks to technological advancements that provide potential improvement in accuracy and safety. Herein, we describe the clinical results of a surgical technique exploiting integration of neuronavigation and intraoperative neurophysiological monitoring (IONM) for minimally invasive PPS.

**Materials and Methods:**

An intraoperative-computed tomography (CT)-based neuronavigation system was combined with IONM in a three-step technique for PPS. Clinical and radiological data were collected to evaluate the safety and efficacy of the procedure. The accuracy of PPS placement was classified according to the Gertzbein–Robbins scale.

**Results:**

A total of 230 screws were placed in 49 patients. Only two screws were misplaced (0.8%); nevertheless, no clinical sign of radiculopathy was experienced by these patients. The majority of the screws (221, 96.1%) were classified as grade A according to Gertzbein–Robbins scale, seven screws were classified as grade B, one screw was classified as grade D, and one last screw was classified as grade E.

**Conclusions:**

The proposed three-step, navigated, percutaneous procedure offers a safe and accurate alternative to traditional techniques for lumbar and sacral pedicle screw placement.

*Level of Evidence* Level 3.

*Trial registration* Not applicable.

## Introduction

Minimal invasive spine surgery has been shown to result in lower rates of complications such as infection and bleeding, reduced postoperative pain, shorter hospitalization, and decreased mortality compared with open surgery [11, 19]. Percutaneous pedicle screw (PPS) placement has been associated with significantly reduced blood loss, duration of surgery, and postoperative pain, as well as faster functional recovery when compared with the traditional open technique [17]. Thanks to technological advancements, image-guided techniques are progressively overcoming the free-hand technique that exclusively depends on anatomical landmarks [12, 24–26].

The traditional free-hand technique needs extensive experience and skill from the surgeon; furthermore, intraoperative fluoroscopy control of the screw placement exposes surgeons and other personnel in the operating room to ionizing radiations. On the other hand, image guidance offers an augmented visualization of the anatomical landmarks without direct exposure. However, there are two major drawbacks: first, the increased radiation exposure for the patient; secondly, the risk of screw malpositioning in case of movement of the reference array. Nevertheless, image-guided screw placement is becoming increasingly popular among spinal surgeons due to the decrease in breach rate and improvements in accuracy [18]. A recent review showed an accuracy rate of pedicle screw positioning of 88.7% using the free-hand technique compared with 96.9% in the image-guided group [28].

Intraoperative neurophysiological monitoring (IONM) is increasingly used in fusion surgery. In particular, triggered electromyographic monitoring is highly specific but weakly sensitive for predicting new postoperative deficit due to pedicle breach during screw placement [20]. In the present paper, we describe a three-step technique that combines a navigated drill guide [based on intraoperative computed tomography (CT) images] with IONM for lumbar and sacral PPS placement. This is, to our knowledge, the first time that the results of the combination of these two technologies have been illustrated.

## Materials and methods

### Patients selection and demographics

Following approval by our local ethical committee (no. 276/120/CE), we conducted a retrospective review of 49 consecutive patients who underwent lumbar arthrodesis with PPS for degenerative conditions of the lumbosacral spine. Surgical procedures were performed by the senior author (G.S.) and his assistant (G.L.R.) and were performed at the same institution. All patients signed a written informed consent before surgery. Inclusion criteria for the study were: (1) age at surgery between 18 and 80 years old, (2) low back pain (LBP) with radicular irradiation in the lower limbs, (3) neurogenic claudication, and (4) failed conservative treatment for at least 6 months. Patients with a previous history of instrumented spine surgery were excluded from the study. Demographic, intraoperative, clinical outcome, and radiological data were recorded.

### Surgical technique

**Fig. 1:**
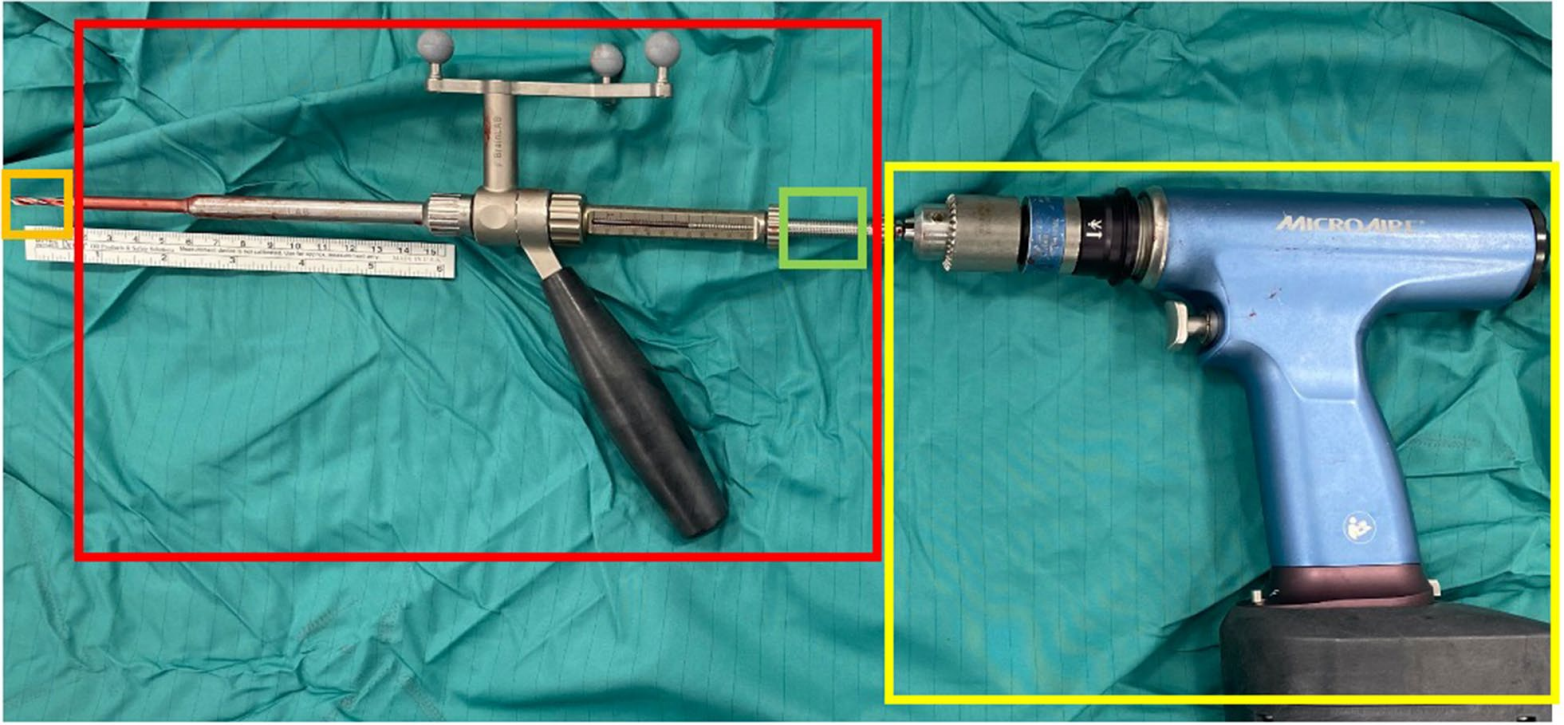
Orange square: the tip of the drill is set to 2.5 cm depth from the entry point. Red square: the navigated guide with hand holder. Green square: the safe screw to set the depth of the drill tip. Yellow square: the low-speed/high-torque power drill

**Fig. 2:**
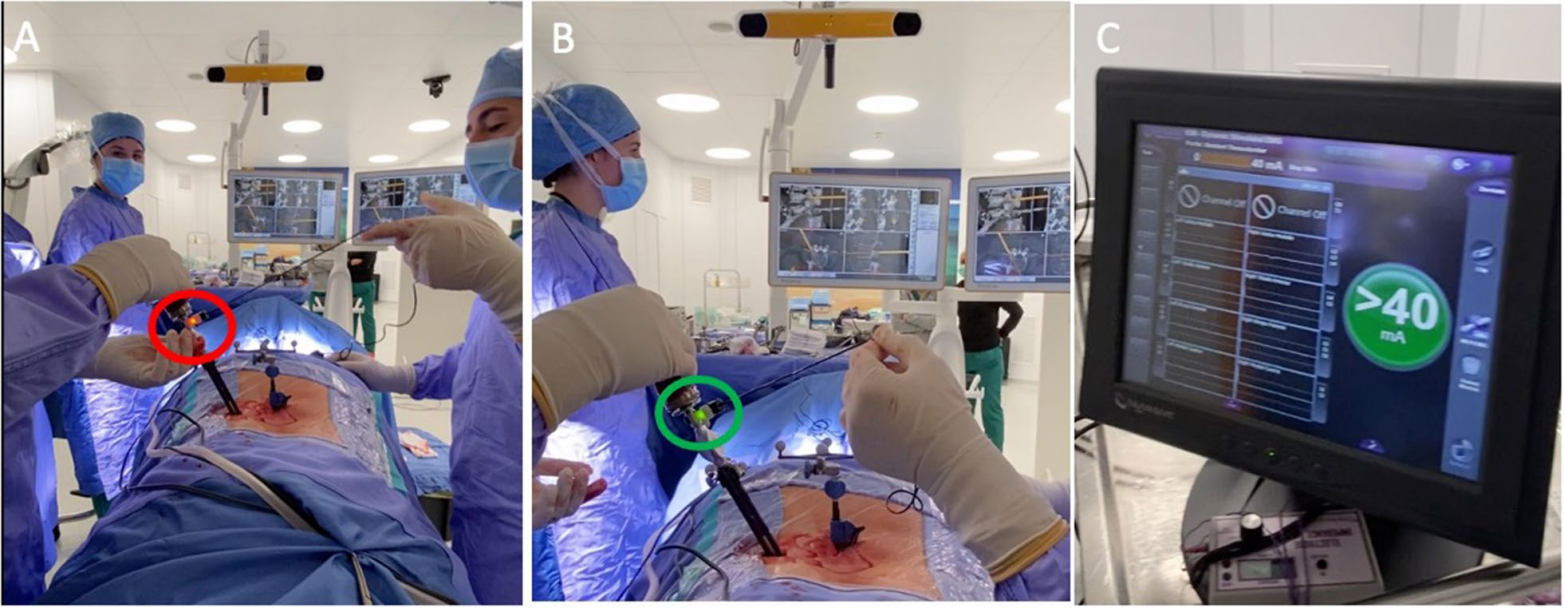
**A** While screwing the pedicle, a red light (red circle) indicates that we are too close to nerve roots. **B** The green light (green circle) indicates no radicular conflicts. **C** The monitor with visual confirmation of the neuromonitoring

**Fig. 3:**
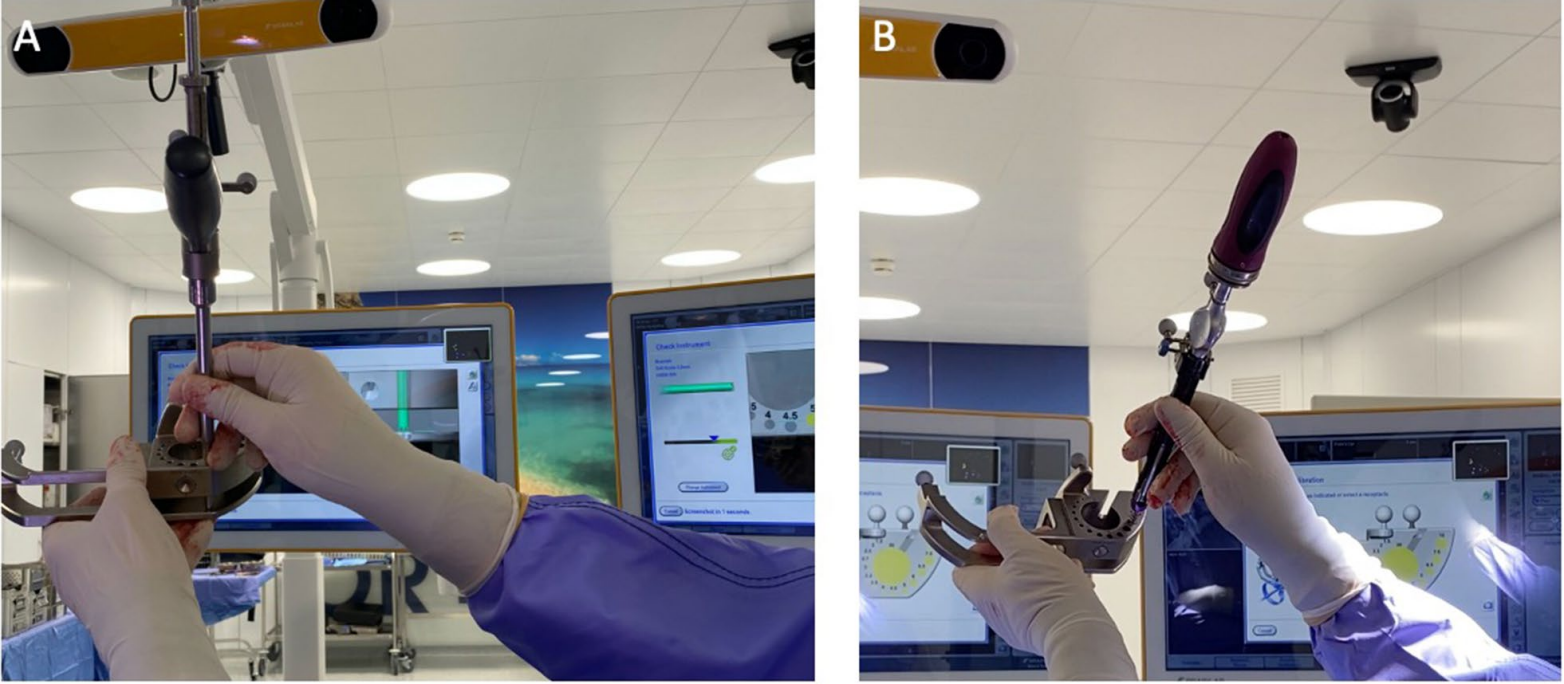
**A** Calibration of the navigated drill guide; **B** calibration of the pedicle screw

**Fig. 4:**
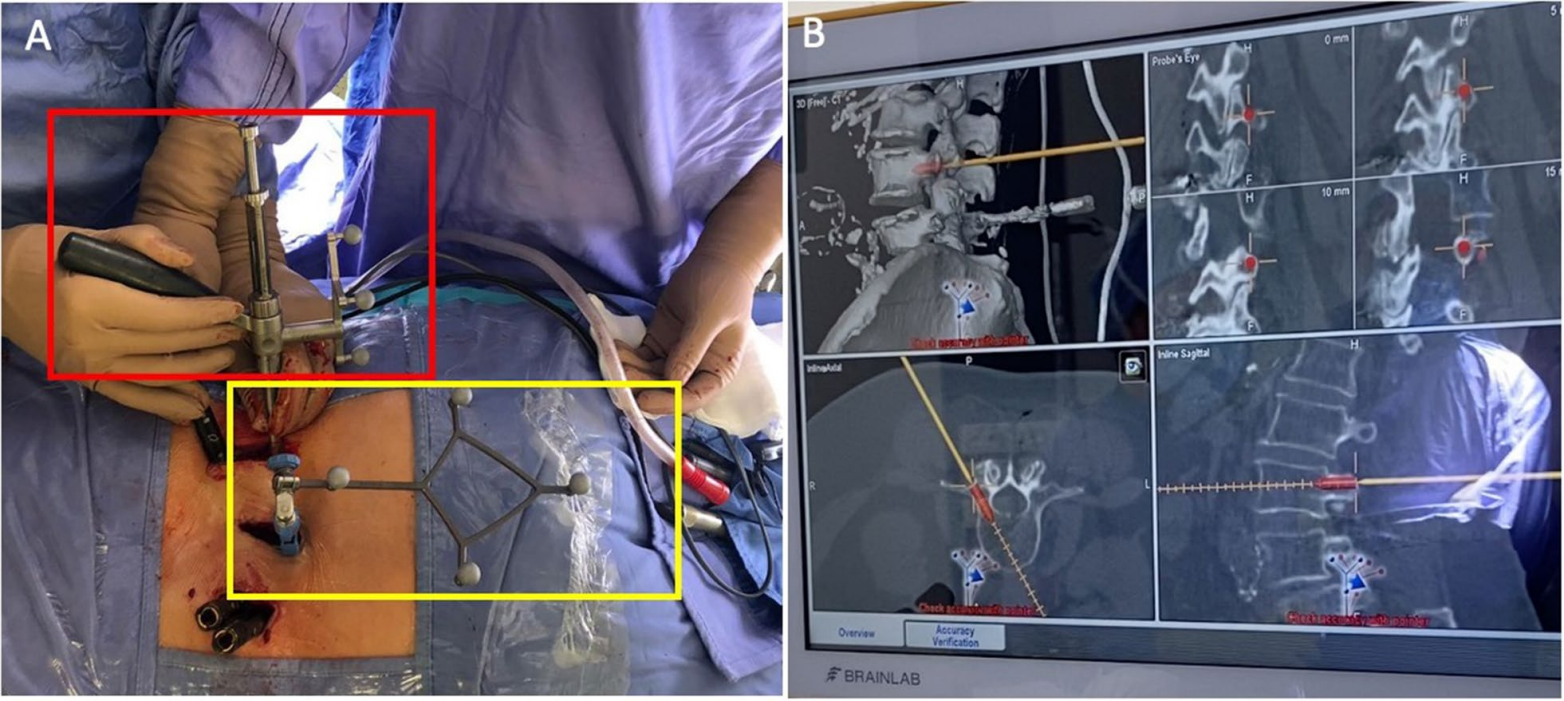
*Step 1*: Placement of the navigated drill guide on the appropriate entry point. **A** Red square: the assistant is holding the navigated drill guide in place while the surgeon inserts the low-speed/high-torque power drill to screw the pedicle. Yellow square: navigation reference clamp of the L5 spinous process. **B** Neuronavigation monitor confirming adequate screw positioning

**Fig. 5:**
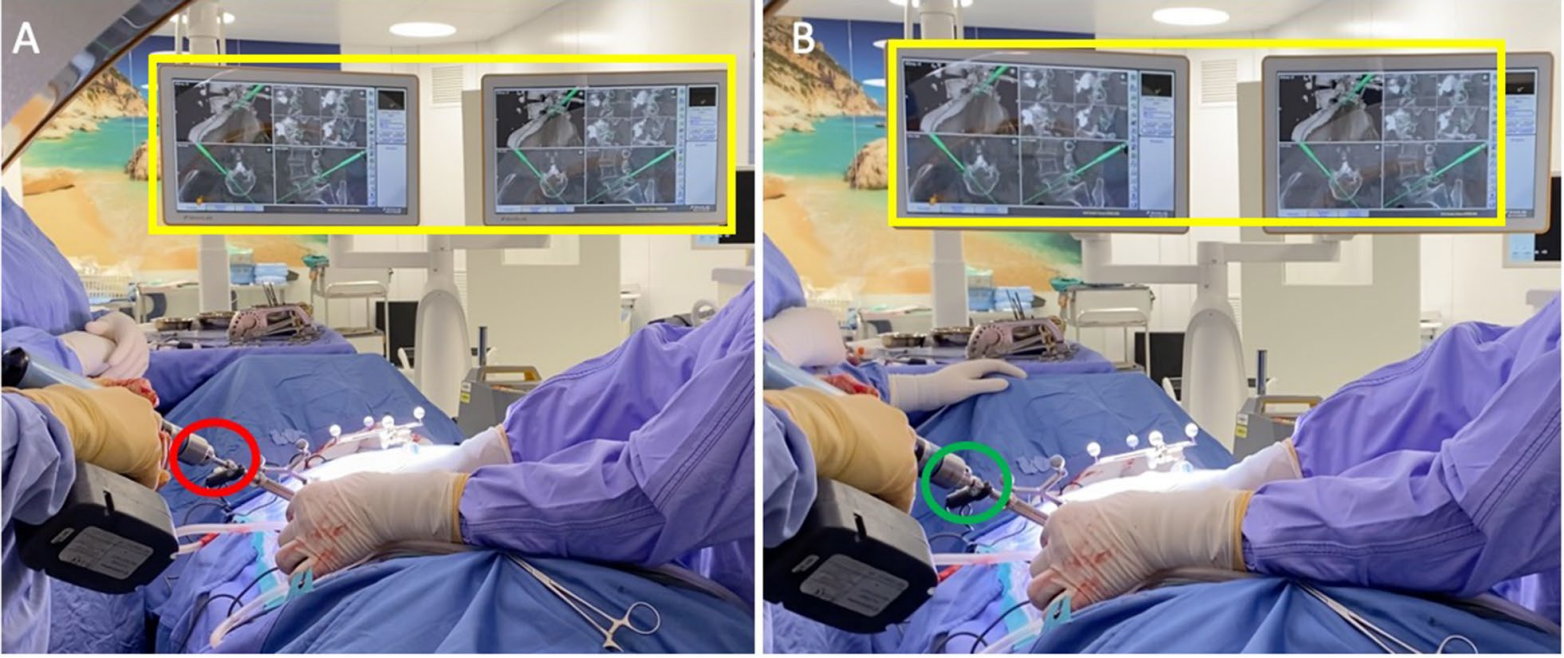
*Step 2*: drilling a hole with the navigated drill guide. The surgeon will handle the drill while the assistant will hold the navigated drill guide. **A** Yellow square: neuronavigated projection we follow with the low-speed/high-torque power drill. Red circle: the length of the drill tip that will enter the pedicle (as seen in **B**, green circle)

**Fig. 6:**
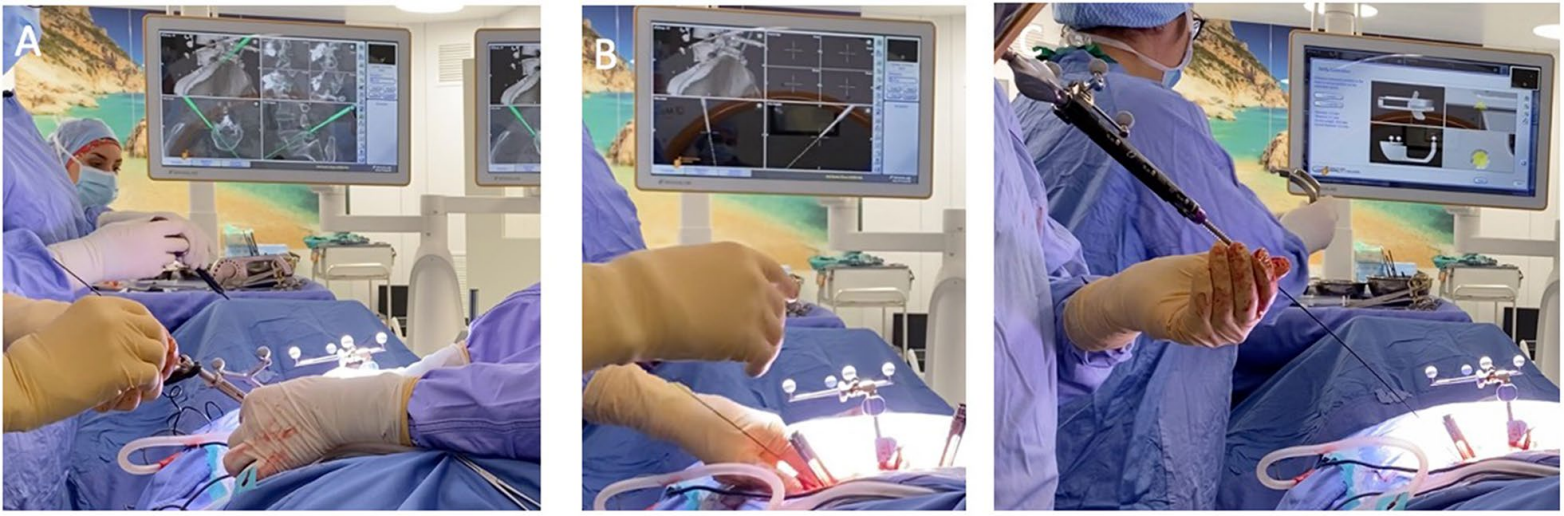
*Step 2.1*: After the drill hole, a k-wire is placed following the same navigated guide direction. **A** The surgeon is placing the K-wire following the navigated drill guide. **B** The surgeon holds the K-wire while the assistant removes the navigated dill guide. **C** Following the K-wire with a navigated screw, the surgeon inserts a screw in the vertebra

**Fig. 7:**
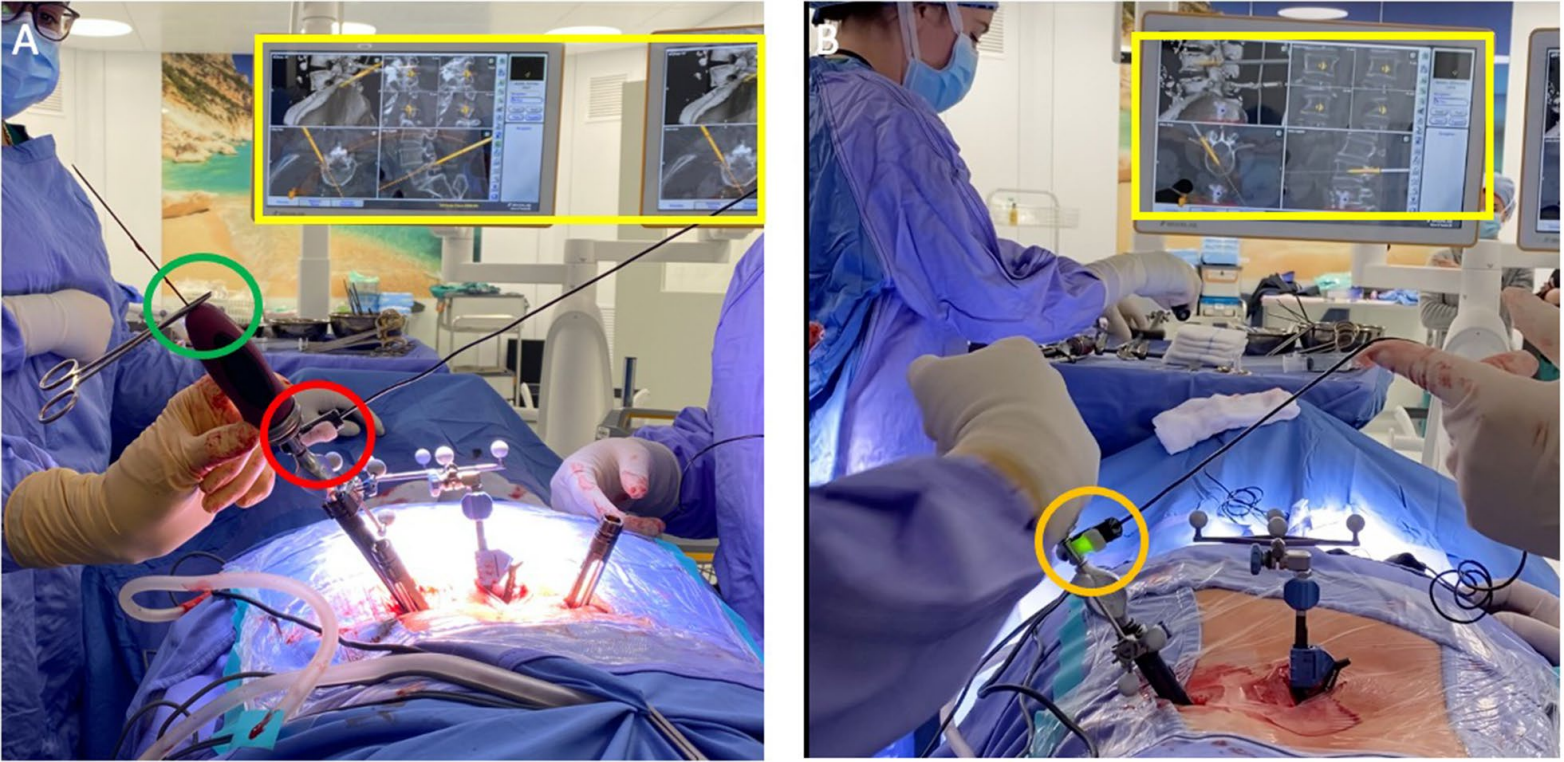
*Step 3*: placement of the pedicle screw. The surgeon will screw the pedicle and vertebral body while the assistant will handle the intraoperative monitoring and will remove the K-wire once the screw enters the vertebral body. **A** Yellow square: real time projection of the screw while it is entering the vertebra. Green circle: Klemmer forceps on the K-wire to be sure that it does not enter together with the screw. Red circle: intraoperative monitoring. **B** Orange circle: the neuromonitoring indicates a green light, meaning no radicular conflict exist

All procedures were performed on a TruSystem 7000 table (TRUMPF Medizin Systeme GmbH) with a BrainLab Curve 1.2 navigation system (Brainlab AG, Munich, Germany) linked to AIRO Mobile intraoperative CT scan (Brainlab AG, Munich, Germany). Instrumentation systems were manufactured by NuVasive (San Diego, California, USA) except for the navigated drill guide (see [6]) and the low-speed/high-torque power drill tip (Fig. 1) that were manufactured by Brainlab. The IONM, Nerve Monitor System (NVM5) was provided by NuVasive and was used for each case (Fig. 2). After prone positioning of the patient, a small lumbar midline incision was performed at the level of the intercristal line (i.e., the line joining the superior aspect of the iliac crests posteriorly). Once a satisfactory exposure of the spinous process was completed, a spinous process bone clamp (Brainlab AG) was tightly attached to the caudal level. The soft tissue retractor was removed before a CT scan to limit artifact of the intraoperative CT and to avoid bone clamp movement. At this point, an intraoperative CT scan was performed, and images were sent to Brainlab Spine & Trauma 3D software (Brainlab AG). Validation of correct levels and adequate image quality was completed by using a pointer. Once the workstation was ready for navigation, the navigated drill guide (Brainlab AG) was verified for accuracy (Fig. 3A) and a small paramedian skin incision (about 1.5 cm) was performed to allow the positioning of the drill guide on the entry point for the pedicle screw (i.e., the junction of the transverse process and lateral facet near the mammillary process) (**step 1**, Fig. 4). A low-speed/high-torque power drill with a 3.5 mm diameter tip was then inserted to perform an entry hole to the depth of 2.5 cm. The depth of drilling was chosen to make sure that the angle of insertion was maintained during screw placement. The Nerve Monitor System (NVM5, NuVasive) was used to rule out any conflict with nerve roots throughout the whole procedure. The appropriate length and diameter of the screw was selected by visualizing a virtual screw projection on the workstation using intraoperative imaging. After the drill was removed (**step 2**, Fig. 5), a Kirshner wire was inserted in the drilled hole. The K-wire was not navigated and must be inserted all the way down to the bottom of the drilled hole. The K-wire can also be used as a feeler to confirm the right access into the pedicle without any breach (Fig. 6). Lastly, the proper cannulated pedicle screw was inserted using the navigated screwdriver, tested for accuracy (Fig. 3B), and placed following the K-wire and the coordinates displayed on BrainLab neuronavigator. Before starting screw insertion, the Nerve Monitor System (NVM5, NuVasive) probe was attached to the screwdriver to make sure no radicular conflict was present. Furthermore, Klemmer forceps were attached to the K-wire to make sure there was no ventral displacement of the wire (**step 3**, Fig. 7). A CT scan was performed at the end of the procedure to confirm the appropriateness of the position of the screws. If necessary, malpositioned screws can be removed and repositioned. A drainage tube was inserted in all cases and removed on postoperative day 1 when the patient is mobilized and discharged home.

### Radiological and clinical outcomes

Age, sex, body mass index (BMI), and length of hospital admission was recorded for all patients. Surgical time between skin incision and first CT scan (which corresponds to the time necessary to place the clamp on the spinous process), and surgical time between first and second intraoperative CT scans (which corresponds to the effective time taken to place pedicle screws) were recorded. Dose exposure was routinely recorded for each patient.

The accuracy of screw placement was evaluated blindly by a senior neuroradiologist on a postoperative CT scan performed the day after the procedure, according to the Gertzbein–Robbins scale [8]. The radiological slice with the largest visible deviation from the pedicle was chosen for grading: a pedicle breach of 2 mm or less was classified as acceptable (grades A and B) while a breach greater than 2 mm (grades C, D, and E) was considered a misplacement.

### Statistical analysis

Statistical analysis was performed using GraphPad Prism 5.01 software (GraphPad Software Inc., San Diego, CA). As appropriate, radiological and clinical data were quantitatively expressed as a range, with relative medians or percentages.

## Results

Forty-nine patients (22 men and 27 women) were treated between January and April 2021 at the same institution during the first 3 months of using the present navigation-guided technique, for a total of 230 screws (208 lumbar and 22 sacral) (Table 1). The median age at surgery was 60 years (range 42–80 years) and the mean length of stay was 2.6 days (range 2–5 days). The most commonly fused segment was L4–L5 (46.9%), followed by L3–L5 (16.3%) and L5–S1 (14.3%) (Table 1).

**Table 1:** Demographic and clinical data of the patient cohort included in the study (*n* = 49)

Demographic/clinical features		Value	%
Age (years)	Median (min/max)	60 (42–80)	
Sex	M/F	22/27	44.8/55.2
BMI (kg/m^2^)	< 25	19	38.8
	25–29.99	12	24.5
	30–34.99	14	28.6
	35–39.99	2	4.1
	> 40	2	4.1
Lenght of stay (days)	Median (min/max)	2.6 (2–5)	
Treated level			
	L2–L3	1	2
	L3–L4	3	6.1
	L4–L5	23	46.9
	L5–S1	7	14.3
	L2–L4	2	4.1
	L3–L5	8	16.3
	L4–S1	4	8.2
	L2–L5	1	2

The average absorbed radiation dose (dose length product) per patient was 1586.4 mGy/cm (range 722.8–2267 mGy/cm). The median time lapse between the two intraoperative CT scans was 29 min (range 59–16 min), corresponding to a total of 6 min and 15 s range (4 min - 14 min and 45 seconds) per screw.

According to the Gertzbein–Robbins scale, only two (0.8%) screws in our series were misplaced (one screw was classified as grade D and one as grade E) (Table 2). The position of the screws was noted at the intraoperative CT scan. Both were laterally displaced, with an “in–out-in” trajectory and no abnormality of IONM. Therefore, the decision was made not to reposition the screws. None of these patients had clinical signs of radiculopathy postoperatively. A perfect trajectory (Gertzbein–Robbins grade A) was observed for 221 of the 230 implanted screws (96.1%). The remaining screws were grades B [(*n* = 7 (3%)], D [*n* = 1 (0.4%)], and E [*n* = 1 (0.4%)] (Table 2).

**Table 2:** Percutaneous pedicle screw placement accuracy according to the Gertzbein–Robbins scale (*n* = 230)

Instrumentation features		Value	%
Total number of screws		230	
Gertzbein–Robbins grade	A	221/230	96.1
	B	7/230	3
	C	0	0
	D	1	0.4
	E	1	0.4
Spinal level			
L2	A	7/8	87.5
	B	1/8	12.5
	C	0	0
	D	0	0
	E	0	0
L3	A	29/32	90.6
	B	2/32	6.3
	C	0	0
	D	1/32	3.1
	E	0	0
L4	A	83/84	98.8
	B	0	0
	C	0	0
	D	0	0
	E	1/84	1.2
L5	A	80/84	95.2
	B	4/84	4.8
	C	0	0
	D	0	0
	E	0	0
S1	A	22/22	100
	B	0	0
	C	0	0
	D	0	0
	E	0	0

## Discussion

The principal aim of this technical note was to report the efficacy and safety achieved with this newly described three-step technique integrating a navigated drill guide and IONM for PPS. In the present study, a perfect route (grade A) was achieved for 221 screws (96.1%) whereas 7 screws (3%) resulted in grade B. Only a minor portion of the screws (0.8%) were graded as D or E, although this did not cause any clinical issue. The accuracy of the described technique is in accordance with literature data relative to image-guided screw placement [3, 10, 14, 21].

Both of our misplaced screws were laterally displaced, with an “in–out–in” trajectory; the mechanical properties of the screws seemed acceptable (as far as it is possible to assess in the operating room). Moreover, the normal IONM response was an adjunctive element that led us to the decision not to replace the screw in both cases. In all cases, including those in which the screws were misplaced, the clinical outcome was uneventful. This is in line with the experience of spinal surgeons and previous literature that not all radiologically misplaced screws lead to clinically relevant consequences [2, 7, 10, 23].

Notably, a long experience with the free-hand technique is very useful to have as a “plan B” technique in case of malfunction of the workstation, and to recognize any eventual mismatch between intraoperative guidance and effective position of the surgical instrument. The tactile feedback of the consistency of the bone is, in fact, an immediate, real-time confirmation that reduces the need of time wasting, repeated checks of accuracy on visible landmarks. In our opinion, the use of intraoperative image-guidance does not replace the need of accurate knowledge of local anatomy and this has a fundamental importance when you decide to try a new strategy or tool. The concomitant use of IONM is an adjunctive source of safety, even though we are aware that there is no definitive evidence that it may prevent neurological deficit [22].

The use of a midline incision for placement of the clamp on the spinous process has not implied an adjunctive incision, as all the patients in our case series underwent lamino-arthrectomy after screw placement. An important issue in this procedure is the exposure to radiation. The patient is exposed to a considerable amount of radiation, even if navigated techniques sometimes showed a lower level of exposure than 2D-fluoroscopy guided procedures [27]. Our level of dose for the patient is similar to procedures already reported in the literature [10, 21]. On the other hand, the reduction in radiation exposure for operating room personnel is also a significant advantage of the described procedure [1, 5, 15, 27].

Several new techniques have been developed in the last few years to improve precision and safety in pedicle screw insertion, such as intraoperative imaging, image-guidance with navigation, robot assistance, and IONM. Though probably not essential, these techniques can help the spinal surgeons, even the most experienced, in reducing risks for the patients. Moreover, they make the training of younger surgeons faster and safer, even if the supervision of an expert in the field is nevertheless fundamental [1, 10, 14]. Wang et al. documented that the O-arm-based navigation is a valid technique that could significantly improve the accuracy of pedicle screw insertion, especially in patients with complex anatomic degenerative diseases. In detail, the operation time in the navigation group was significantly less than that in the free-hand group and the accuracy rate of pedicle screw positioning was 88.7% in the free-hand group and 96.9% in the O-arm group [13]. Our results, in terms of accuracy, are in line with previous literature regarding navigated PPS placement [16, 21].

A recent meta-analysis compared the revision rate of PPS in free-hand, navigated, and robot-guided thoracolumbar instrumentation, and showed that assisted techniques (robotic or navigated) may reduce the incidence of costly and clinically relevant postoperative revisions for screw malposition [23].

Our experience with the described technique highlighted some important points to improve the safety and accuracy of the procedure:

- 1. It is of paramount importance to secure the spinous process clamp before the CT scan. The clamp must be tightly fixed on the bone, not to the ligament. At the same time, it is necessary not to use too much force to avoid fractures of the spinous process or breaking the clamp. The soft tissue retractor is removed before the CT scan to reduce artifacts. The reference attached to the clamp must not be in contact with the skin and should have the correct inclination to be visible both at the moment of the CT scan and during screw placement. Moreover, it must not hamper the screw placement. The use of the drill guide instead of a navigated trocar has the advantage of reducing the vibrations that are transmitted to the clamp, with the risk of unintentionally moving the reference.
- 2. IOM helps to avoid neurologically significant misplacement of the screw and gives a continuous, real-time feedback during pedicle drilling and during screw placement. An intraoperative CT scan performed after screw placement provides an immediate control of the position of the screws relative to the anatomy of the patient. The combination of neurophysiological and radiological data improves the safety of the procedure.
- 3. This technique allows the placement of only one screw at a time, but the collaboration between the two operating surgeons and the standardization of the workflow greatly reduces the time and the risk of inaccuracy.
- 4. Cooperation between surgeons is essential during placement of the Kirschner wire and for removal of the drill guide. This is a crucial step, usually done with four hands because it is the only “blind” step of the procedure. The risk of skiving on the bony surface, especially in case of facet bony spurs, is lowered by the presence of sharp prominences on the extremity of the drill guide, but the collaboration between surgeons remains the most important element in avoiding mistakes. The wire is inserted only 2.5 cm deep in the pedicle, as set with the drill guide, so the real risk of penetration of the wire in the abdominal viscera and major blood vessels is very low.
- 5. Finally, usage of a K-wire has, in our opinion, two advantages: (a) the wire can be used as a feeler to confirm the bony consistence of the tissue before screw placement and (b) in case of inaccuracy of image guidance, the immediate placement of the screw in an incorrect position has a higher risk of irreversible damage to neural structures or to pedicle.

Our study/technique has some limitations. The present study was conducted on a small number of patients and only on degenerative lumbo–sacral pathology. There is a risk of skiving on the drill guide during K-wire insertion [9]. The risk is higher in cases where facet bony spurs are present. This drawback does not affect the open technique. Finally, the ergonomics of the low-speed/high-torque power drill needs four hands to drill the pedicle.

In conclusion, this study suggests that our three-step technique for PPS can be a valid option to use in minimally invasive spine surgery (MISS) procedures. It exploits advantages of neuromonitoring and CT-guided navigation. We have used this technique for implantation of more than 200 pedicle screws with no complications so far, and excellent results in terms of pedicle screws placement accuracy.

## Data Availability

The datasets generated during the current study are available from the corresponding authors upon request.

## Abbreviations

MISS: Minimally invasive spine surgery
PPS: Percutaneous pedicle screws
BMI: Body mass index
CT: Computed tomography
